# Thyroid hormone treated astrocytes induce maturation of cerebral cortical neurons through modulation of proteoglycan levels

**DOI:** 10.3389/fncel.2013.00125

**Published:** 2013-08-12

**Authors:** Rômulo S. Dezonne, Joice Stipursky, Ana P. B. Araujo, Jader Nones, Mauro S. G. Pavão, Marimélia Porcionatto, Flávia C. A. Gomes

**Affiliations:** ^1^Instituto de Ciências Biomédicas, Universidade Federal do Rio de JaneiroRio de Janeiro, Brazil; ^2^Instituto de Bioquímica Médica and Hospital Universitário Clementino Fraga Filho, Universidade Federal do Rio de JaneiroRio de Janeiro, Brazil; ^3^Departamento de Bioquímica, Universidade Federal de São PauloSão Paulo, Brazil

**Keywords:** astrocyte, thyroid hormones, neurite outgrowth, extracellular matrix, brain morphogenesis

## Abstract

Proper brain neuronal circuitry formation and synapse development is dependent on specific cues, either genetic or epigenetic, provided by the surrounding neural environment. Within these signals, thyroid hormones (T3 and T4) play crucial role in several steps of brain morphogenesis including proliferation of progenitor cells, neuronal differentiation, maturation, migration, and synapse formation. The lack of thyroid hormones during childhood is associated with several impair neuronal connections, cognitive deficits, and mental disorders. Many of the thyroid hormones effects are mediated by astrocytes, although the mechanisms underlying these events are still unknown. In this work, we investigated the effect of 3, 5, 3′-triiodothyronine-treated (T3-treated) astrocytes on cerebral cortex neuronal differentiation. Culture of neural progenitors from embryonic cerebral cortex mice onto T3-treated astrocyte monolayers yielded an increment in neuronal population, followed by enhancement of neuronal maturation, arborization and neurite outgrowth. In addition, real time PCR assays revealed an increase in the levels of the heparan sulfate proteoglycans, Glypican 1 (GPC-1) and Syndecans 3 e 4 (SDC-3 e SDC-4), followed by a decrease in the levels of the chondroitin sulfate proteoglycan, Versican. Disruption of glycosaminoglycan chains by chondroitinase AC or heparanase III completely abolished the effects of T3-treated astrocytes on neuronal morphogenesis. Our work provides evidence that astrocytes are key mediators of T3 actions on cerebral cortex neuronal development and identified potential molecules and pathways involved in neurite extension; which might eventually contribute to a better understanding of axonal regeneration, synapse formation, and neuronal circuitry recover.

## Introduction

Central nervous system (CNS) development is characterized by differentiation of neural progenitors into different cell types, proper neuronal migration and maturation, and axonal growth in order to innervate specific targets. Accuracy in neuronal circuitry formation and synapse development is dependent on specific cues provided by the surrounding neural environment, either genetic or epigenetic (Powell et al., 1997). Astrocytes, the most abundant glial cell in the CNS, are source of most the extracellular matrix components (ECM) and neurotrophic factors involved in these events, including 3, 5, 3′-triiodothyronine (T3), the biological active form of thyroid hormones, which are essential morphogens of the nervous system (Garcia-Abreu et al., 1995; Trentin et al., 1995; Ullian et al., 2001, 2004; Martinez and Gomes, 2002; Christopherson et al., 2005; Lie et al., 2005; Martinez and Gomes, 2005; Kornyei et al., 2007; Spohr et al., 2008; Barker and Ullian, 2010; Stipursky et al., 2011, 2012; Tc et al., 2011).

Thyroid hormones, thyroxin (T4) and T3, influence critical events in brain morphogenesis including neuronal migration and differentiation, glial cells maturation, synaptogenesis, and myelination (Alvarez-Dolado et al., 1999; Auso et al., 2004; Cuevas et al., 2005; De Escobar et al., 2007; Dezonne et al., 2009; Portella et al., 2010). Hypothyroidism leads to impaired cerebral cortical layering and altered callosal connections (Gravel et al., 1990; Berbel et al., 1993; Calikoglu et al., 1996). Moreover, it has been associated with clear reductions on axonal and dendritic outgrowth (Eayrs, 1955; Ruiz-Marcos et al., 1979) as well as disorganization of dendritic spines and fewer synaptic connections (Ruiz-Marcos et al., 1988) in experimental models. In humans, insufficient levels of thyroid hormones during brain development are associated with severe neurological deficits that might result in the cretinism syndrome (Joffe and Sokolov, 1994).

T4 is the predominant hormone form found in the nervous tissue, where it is converted to the active form, T3, by the type II iodothyronine 5′-deiodinase (D2), mainly expressed by astrocytes and tanycytes (Guadano-Ferraz et al., 1997; Santisteban and Bernal, 2005). Activation of major signaling pathways by T3/T4 involves binding of T3 to nuclear receptors, although extranuclear pathways have also been described (Santisteban and Bernal, 2005; Bernal, 2007). Thyroid hormones nuclear pathway is triggered by activation of thyroid hormone receptors alpha (TRα) and beta (TRβ) widely expressed by neuronal and glial cells (Puymirat, 1992; Carlson et al., 1996; O'Shea and Williams, 2002; Bernal, 2007).

Despite thyroid hormones effects on neurons are well known, several works have shown that some of these effects might be indirectly controlled by astrocytes (Trentin and Moura Neto, 1995; Gomes et al., 1999; Martinez and Gomes, 2002, 2005).

Thyroid hormones have been reported to modulate astrocyte morphology, differentiation, and proliferation (Lima et al., 1997; Trentin et al., 1998; Trentin, 2006), and to regulate ECM organization and synthesis (Farwell and Dubord-Tomasetti, 1999a,b; Calloni et al., 2001; Martinez and Gomes, 2002; Mendes-De-Aguiar et al., 2008). *In vivo*, thyroid hormones regulate radial glia-astrocyte transition and the vimentin- glial fibrillary acidic protein (GFAP) switch, a hallmark of astrocyte differentiation, in the basal forebrain and hippocampus (Gould et al., 1990; Martinez-Galan et al., 1997, 2004). Although astrocytes are evident targets for thyroid hormones *in vitro* and *in vivo*, the precise effects of these hormones in neuron-astrocyte interactions are still under investigation.

We previously demonstrated that thyroid hormone-treated cerebellar astrocytes induce cerebellar progenitor proliferation (Gomes et al., 1999) and neurite outgrowth (Martinez and Gomes, 2002, 2005). These events result from the synthesis and secretion of soluble factors, such as epidermal growth factor (EGF), and the ECM molecules laminin (LN), fibronectin (FN) and syndecans, producing a substrate for neuronal maturation (Mendes-De-Aguiar et al., 2008, 2010).

In the present work, we investigated the role of cortical astrocytes as mediators of thyroid hormone T3, on neuronal maturation, using an *in vitro* system consisting of astrocyte-neuron cocultures. Here we report that thyroid hormone-primed astrocytes increase neuronal differentiation and neuritic arborization, mainly by modulation of chondroitin and heparan sulfate proteoglycans (HSPG).

## Materials and methods

### Ethical approval

All animal protocols were approved by the Animal Research Committee of the Federal University of Rio de Janeiro (DAHEICB024).

### Astrocyte primary culture

Astrocytes primary cultures were prepared from cerebral cortex derived from newborn Swiss mice, as previously described (Spohr et al., 2008). Briefly, after mice decapitation, brain structures were removed and the meninges were carefully stripped off. Tissues were washed in phosphate-buffered saline (PBS), 0.6% glucose (Merck, Darmstadt, Hessen, DE) and cortical structures were dissociated into single cells in a medium consisting of Dulbecco's modified Eagle's medium supplemented with nutrient mixture F-12 (DMEM/F-12, Invitrogen Life Technologies, Carlsbad, California, USA), enriched with glucose (3.3 × 10^−2^ M), glutamine (2 × 10^−3^ M) and sodium bicarbonate (0.3 × 10^−2^ M). Dissociated cells were plated onto plastic culture flask or glass cover slips (24 wells plates, Techno Plastic Products, Trasadingen, CH) previously coated with polyornithine (1.5 μg/mL, molecular weight 41,000; Sigma Chemical Co., St Louis, Missouri, USA) in DMEM/F12 supplemented with 10% fetal bovine serum (FBS) (Invitrogen). The cultures were incubated at 37°C in a humidified 5% CO_2_, 95% air chamber. After 24 h, cell cultures were washed and media were replaced by DMEM/F-12 supplemented with 10% FBS. The medium was changed every second day until reaching confluence.

### T3 treatment and conditioned medium (CM) preparation

After reaching confluence, glial monolayers were washed three times with serum-free DMEM/F12 medium, and incubated for an additional day in serum-free medium. After this period, cultures were treated with 50 nM of T3 (Sigma Aldrich) in DMEM/F12 for 3 days with daily medium change. Control astrocyte carpets were maintained in DMEM/F12 without serum. After that, glial monolayers were washed three times with serum-free DMEM/F12 and maintained for an additional day with serum-free medium. CMs derived from T3-treated (T3-CM) or control cultures (C-CM) were recovered, centrifuged at 1500 g for 10 min, and used immediately or stored at −70°C for further use.

### Enzymatic treatment of astrocyte monolayers

To analyze a possible influence of glycosaminoglycans, astrocyte monolayers were enzymatically digested with chondroitinase AC (5.0 × 10^−7^ U/μL) (that specifically digests chondroitin sulfate glycosaminoglycan chains) or heparanase III (5.0 × 10^−7^ U/μL) [that specifically digests heparan sulfate glycosaminoglycan (HSG) chains] (Sigma Aldrich) in DMEM/F12, for 2 h at 37°C, prior to addition of progenitor cells. After, cultures were extensively washed with medium without serum to remove all residual enzymes, followed by addition of neuronal progenitors to astrocyte monolayers.

### Neural progenitor culture and astrocyte-neural progenitor coculture

Pregnant Swiss females with 14-gestational days were killed by halothane followed by cervical dislocation, and embryos (E14) were removed. Cortical progenitors were prepared as previously described (Spohr et al., 2008). Briefly, for coculture assays cells were freshly dissociated from cerebral cortex and 5 × 10^4^ cells were plated onto control, thyroid hormones-treated glial monolayer carpets or onto astrocyte-carpets previously digested with chondroitinase or heparanase III. Cocultures were kept for 24 h at 37°C in a humidified 5% CO_2_, 95% air atmosphere. For pure neural progenitor cultures 1 × 10^5^ dissociated cells were plated onto glass cover slips previously coated with polyornithine, and incubated with C-CM or T3-CM. Cultures were kept for 24 h at 37°C in a humidified 5% CO_2_, 95% O_2_ air atmosphere.

### Immunocytochemistry

Cells were fixed with 4% paraformaldehyde for 5 min for ECM protein analyses or 15 min, for cytoskeleton protein analyses. For cytoskeleton proteins analysis, cells were additionally permeabilized with 0.2% Triton-X (Vetec Química Fina Ltda, Rio de Janeiro, Rio de Janeiro, BR) for 5 min at room temperature. Subsequently, cells were blocked with 5% bovine serum albumin (Invitrogen) and 3% normal goat serum (Invitrogen) in PBS (block solution) for 1 h and incubated overnight at 4°C with the specified primary antibody diluted in block solution. Primary antibodies were mouse anti−βTubulin III antibody (Promega Corporation; Madison, Wisconsin, USA; 1:1000); rabbit anti-GFAP (Dako, Glostrup, DK; 1:500); rabbit anti-Fibronectin (Sigma Aldrich; 1:200); rabbit anti-Laminin (Sigma Aldrich; 1:100). After primary antibodies incubation, cells were extensively washed in PBS and incubated with the following secondary antibodies diluted in block solution for 2 h: goat anti-mouse IgG conjugated with Alexa Fluor 488 or goat anti-rabbit IgG and anti-rat IgG conjugated with Alexa Fluor 546 (Molecular Probes, Eugene, Oregon, USA; 1:400, 1:500, and 1:1000, respectively). Cell nuclei were labeled with 4′,6-diamidino-2-phenylindole dihydrochloride (DAPI) and cell preparations were mounted directly on N-propyl gallate (Sigma Aldrich). Negative controls were obtained by omitting primary antibodies; in all cases, no reactivity was observed. After immunostaining, cell cultures were visualized and counted using a TE300 Nikon microscope. At least 10 fields were counted per well.

### Neuronal morphometry

To analyze neurite outgrowth, neuronal cells cultured either onto astrocyte monolayers or onto glass cover slips, were measured using the NeuronJ plug-in of Image J 1.36 b software. At least 10 fields were measured per well. In all cases, at least 100 neurons randomly chosen were observed per well. All neurites emerged from neuronal soma were considered. Neurite length was analyzed by 3 different methods either considering only the major process per neurons, the sum of all neurite measurements per neuron and the sum of all neurite measurements divided by the number of process per neuron.

### Western blot

Protein concentration on cell extracts was measured by the BCA™ Protein Assay Kit (Pierce, IL, USA). Fifty micrograms of protein per lane were eletrophoretically separated in 5–15% gradient sodium dodecyl sulfate-polyacrylamide gel (SDS-PAGE). After separation, proteins were electrically transferred onto a Hybond-P polyvinylidene difluoride transfer membrane (Amersham Biosciences, Little Chalfont, Buckinghamshire, UK) for 3 h. Membranes were blocked overnight in Tris-buffered saline-Tween 20 (Merck) (TBS-T) containing 10% BSA. Primary antibodies were added for 2 h at room temperature. After several washes in TBS-T, peroxidase-conjugated secondary antibodies were added to membrane and incubated for 2 h at room temperature. Proteins were visualized using the enhancing chemiluminescence detection system (Super Signal West Pico Chemiluminescent Substrate/Pierce, Milwaukee, Wisconsin, USA), and PVDF membranes were exposed to autoradiographic films (Kodak, São José dos Campos, São Paulo, BR). Primary antibodies were mouse anti−α-Tubulin (Sigma Aldrich; 1:5000); rabbit anti-Fibronectin (Sigma Aldrich; 1:1000); rabbit anti-Laminin (Sigma Aldrich; 1:1000). Secondary peroxidase-conjugated antibodies were goat anti-rabbit IgG and goat anti-mouse IgG (Amersham Biosciences; 1:5000). After protein detection, densitometric analysis of autoradiographic films was done using Image J 1.36 b software.

### RT-PCR

Total RNA was isolated from cells using TRIZOL (Invitrogen) according to the protocol provided by the manufacturer. After DNAse treatment (RQ1 RNAse-free DNAse, Promega Wisconsin, USA), RNA samples (up to 1.5 μg) were reverse-transcribed into cDNA using oligo (dT) and Super ScriptTM II Reverse transcriptase (Invitrogen). cDNA was amplified by Taq DNA Polymerase in 103 PCR Buffer using Invitrogen's protocol. Sense and antisense specific oligonucleotides were in Table 1. Amplification was performed in 35 cycles, and PCR products were size-fractionated by electrophoresis using a 2% agarose gel and visualized by ethidium bromide staining. Negative controls for genomic DNA contamination were carried out. Densitometries were done using Image J 1.36 b software.

**Table 1:** **Specifications of the oligonucleotides used in conventional and real time RT-PCR**.

**Gene**	**Sequence**	**Product (bp)**
GFAP	5′ CGA TTC AAC CTT TCT CTC CAA ATC CAC ACG 3′	339
	5′ CTT TGC TAG CTA CAT CGA GAA GGT CCG CTT 3′	
TRα1	5′ GGT GCT GCA TGG AGA TCA TG 3′	225
	5′ GGA ATG TTG TGT TTG CGG TG 3′	
TRβ1	5′ CGG AGG AGA AGA AAT GTA AAG G 3′	421
	5′ GCT TCG GTG ACA GTT TTG AT G 3′	
Dio2	5′ CTT GAC TTT GCC AGT GCA GA 3′	351
	5′ GCA CAC ACG TTC AAA GGC TA 3′	
GAPDH	5′ AAG AAG GTG GTG AAG CAG GCA TCT 3′	200
	5′ ACC CTG TTG CTG TAG CCG TAT TCA 3′	
Syndecan-3	5′ TCG TTT CCT GAT GAT GAA CTA GAC 3′	301
	5′ GTG CTG GAC ATG GAT ACT TTG TT 3′	
Syndecan-4	5′ AGA GCC CAA GGA ACT GGA AGA GAA 3′	147
	5′ ATC AGA GCT GCC AAG ACC TCA GTT 3′	
Glypican-1	5′ ACT CCA TGG TGC TCA TCA CTG ACA 3	151
	5′ TTT CCA CAG GCC TGG ATG ACC TTA 3′	
Versican	5′ TCC AGG AGA AAC AGT TGG GAT GCT 3′	192
	5′ AAG GAA GGA AAG GTT GGC CTC TCA 3′	

### Quantitative RT-PCR

Total RNA was Trizol® (Invitrogen, USA) extracted from astrocytes monolayers were used for RNA extraction and RNA was quantified using NanoDrop ND-1000 Spectrophotometer (Thermo Fisher Scientific, USA). Two micrograms of total RNA were reverse-transcribed with Oligo (dT) 15 Primer and ImProm-II Reverse Transcription System with Recombinant RNasin® Ribonuclease inhibitor (Promega, USA) and MgCl2. The primer sequences were verified to be specific using GenBank's BLAST (Altschul et al., 1997). The primers used in this assay were in Table 1. Quantitative real-time RT-PCR was performed using SYBR®-Green PCR Master Mix, including AmpliTaq-GOLD polymerase (Applied Biosystems, USA). Reactions were performed on ABI PRISM 7500 Real Time PCR System (Applied Biosystems). The relative expression levels of genes were calculated using the 2−Δ ΔCT method (Livak and Schmittgen, 2001). The amount of target genes expressed in a sample was normalized to the average of the endogenous control. This is given by ΔCT, where ΔCT is determined by subtracting the average endogenous gene CT value from the average target gene CT value [CT target gene—CT average (endogenous gene)]; where 2−ΔCT is the relative expression of the target gene compared to the endogenous gene. The calculation of Δ ΔCT was done by subtracting ΔCT value for the controls from the ΔCT value for a given treatment [ΔCT target gene (treated)—ΔCT target gene (control)]; where 2−Δ ΔCT is the relative expression of the target gene at T3-treated astrocytes compared to controls.

### Enzyme-linked immunosorbent assays (ELISA)

A quantitative indirect immunoenzyme assay was performed after the protein levels were measured. Polystyrene microtiter plate wells (Maxisorp, Nunc, and Roskilde, Denmark) were coated with 50 μL of protein (5 μg/mL in PBS) by passive adsorption overnight at 4°C. The plates were then washed with PBS containing 0.05% Tween 20 and 0.1% BSA (PBS-Tween). Non-specific binding was blocked by incubating the plates for 2 h with 1% BSA in PBS, pH 7.4 at 37°C. After an additional PBS-Tween washing, the plates were incubated with the primary antibodies, rabbit anti-Fibronectin (Sigma Aldrich; 1:1000); rabbit anti-Laminin (Sigma Aldrich; 1:1000) and mouse anti−α-Tubulin (as control) (Sigma, USA; 1:3000) for 24 h at 4°C, followed by incubation with a goat anti-rabbit or mouse IgG peroxidase-linked conjugated antibody (1:8000 Amersham Biosciences, UK). The plates were washed with PBS-Tween, and the reaction was developed with the substrate o-phenylenediamine (0.5 mg/mL and 0.005% H_2_O_2_ in 0.01 M sodium citrate buffer, pH 5.6) (Vetec, Brazil). The reaction was stopped with 0.2 M H_2_SO_4_ (Vetec, Brazil), and the absorbance was measured using an automated reader (BioRad ELISA Reader, Hercules, CA, USA). The experimental groups consisted of triplicate samples from three independent experiments.

### Statistical analyses

Statistical analyses were done using one-way non-parametric ANOVA coupled with Tukey post-test by GraphPad Prism 4.0 software, and *P* < 0.05 was considered statistically significant. The experiments were performed in triplicate, and each result represents the mean of at least four independent experiments.

## Results

### Cerebral cortex astrocytes primed by T3 induce neuronal fate commitment and enhance neuronal maturation

**Figure 1:**
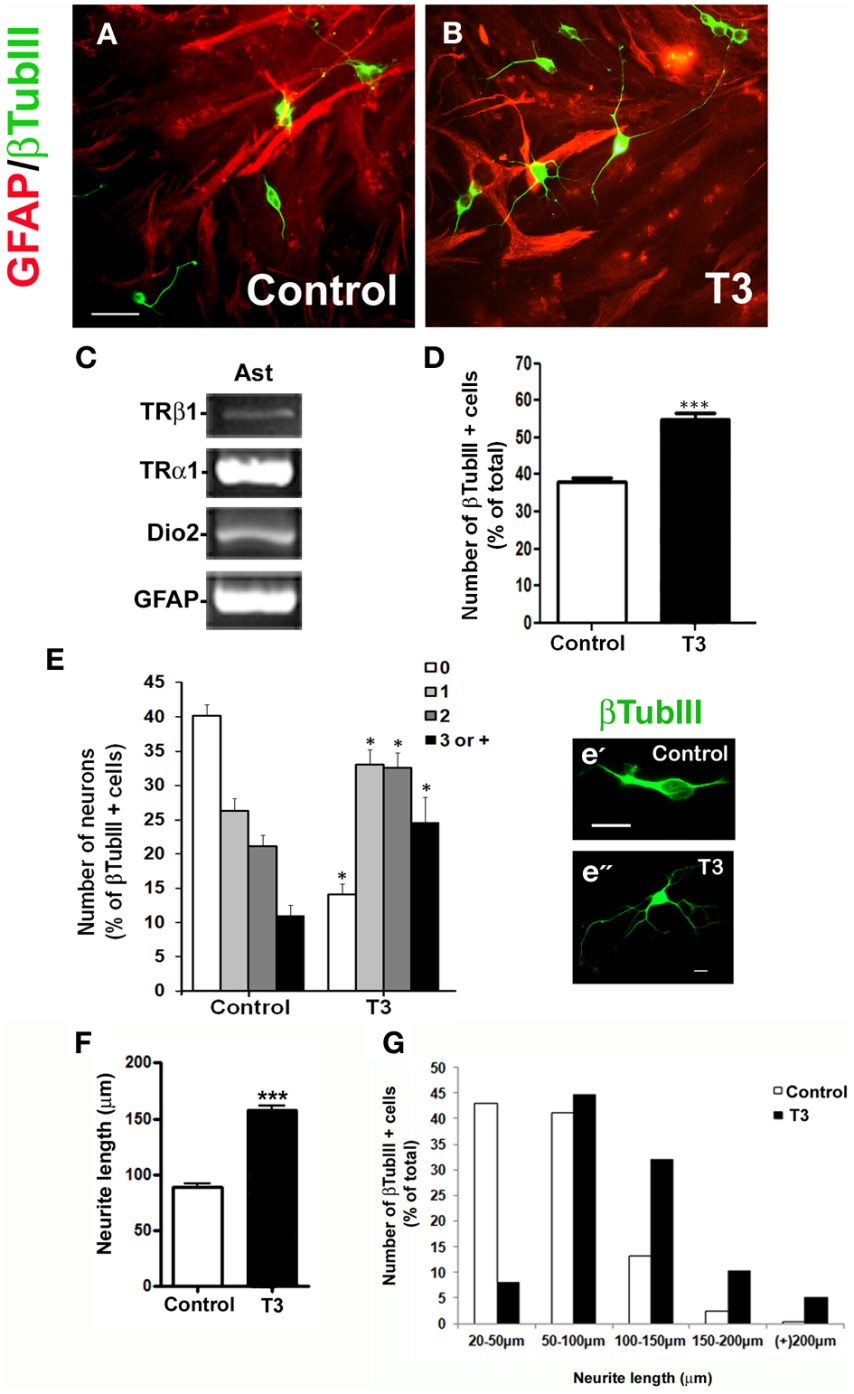
**Thyroid hormone-primed astrocytes induce neuronal differentiation and arborization:** Cortical progenitors obtained from E14 mice were cultured onto untreated (**A**; Control) and T3 **(B)** astrocyte monolayers. After 24 h of coculture, neurons were morphologically characterized by βtubulin III (βtubIII+) immunostaining for number of neurons **(D)**, number of neurites **(E)** and length of neurites **(F,G)**. In all cases, at least 100 neurons randomly chosen were observed. RT-PCR assays identified expression of members of thyroid hormone signaling pathway in cultured cortical astrocytes: TRα1, TRβ1, and D2 mRNAs **(C)**. Note the complexity of neuronal cells plated over T3-astrocytes **(e”)** compared with control-astrocytes **(e')**. Increase in arborization complexity was also followed by an increment in neurite length **(G)**. Scale bars: 50 μm **(A)**, 10 μm **(e')**. ^*^P < 0.05; ^***^P < 0.001.

In order to evaluate the role of astrocytes as mediators of T3 in cerebral cortex neuron development, astrocyte monolayers derived from newborn cerebral cortex were primed by 50 nM of T3 for 3 days, followed by coculture with embryonic cerebral cortex progenitors for 24 h (Figures 1A,B).

RT-PCR assays of control astrocyte monolayers demonstrated that astrocytes express both TRα1 and TRβ1 receptors, as well type II deiodinase (D2) mRNAs, thus, ensuring that they might respond to thyroid hormone (Figure 1C).

Culture of progenitor cells onto T3-primed astrocyte monolayers yielded a 44% increase in neuronal population as revealed by immunostaining for the neuronal marker βTubulinIII (Figure 1D). Morphometric analysis revealed an 80% increase in the number of neurons with three or more processes, followed by a 62% decrease of aneuritic cells, when neurons were cultured onto T3-primed astrocyte carpets (Figure 1E).

In order to evaluate the effect of thyroid hormones-treated astrocytes on axonal growth, neurite length was analyzed by three parameters: either considering the sum of total neurite length per neuron; the longest neurite per neuron or the sum of all neurite measurements divided by the number of process per neuron. Treatment of astrocytes by T3 promoted a 75% increment on neurite outgrowth (Figure 1F). Moreover, around 33% of neuronal cells plated over astrocyte monolayers treated with thyroid hormones developed neurites with an average size around 100–150 μm whereas less than 15% of neurons plated over control astrocytes displayed these characteristics (Figure 1G). Treatment of astrocytes by T3 greatly decreased the number of neurons with neurites under 50 μm (control: 23% × T3-treated: 1%) and increased those bigger than 150 μm (control: 2% × T3-treated: 10–18%) (Figure 1G).

Together, these results show that thyroid hormone induce astrocytes to adopt a permissive and/or inductive phenotype that favors neuronal fate specification of cortical progenitors followed by further neuronal maturation and arborization.

### Effect of astrocytes soluble factors on neuronal differentiation and maturation

**Figure 2:**
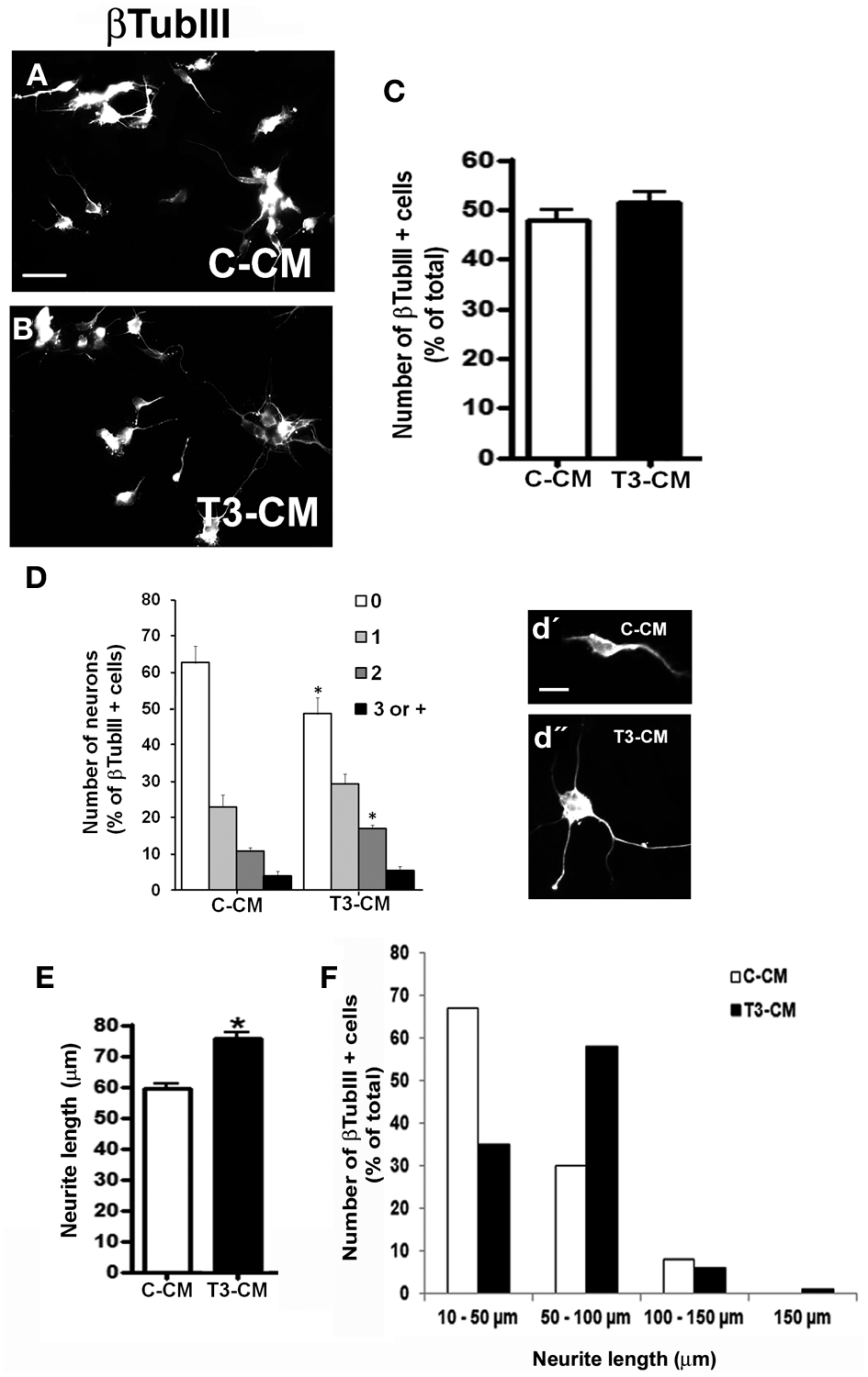
**Astrocytic soluble factors secreted in response to thyroid hormones affect neuronal differentiation.** E14 cerebral cortex progenitors were cultured in the presence of control conditioned medium obtained from non-treated astrocytes (**A**; C-CM) and T3-treated astrocytes (**B**; T3-CM) for 24 hr. Neurons were morphologically characterized by βtubulin III (βtubIII+) immunostaining for number of neurons **(C)**, number of neurites **(D)** and length of neurites **(E,F)**. Note that although T3-CM enhanced neuronal arborization and neurite outgrowth **(d”)**, it did not affect number of neurons **(C)**. Scale bars: 50 μm **(A)**, 10 μm **(d')**. ^*^P < 0.05.

Astrocytes constitute the main source of neurotrophic factors during nervous system development (Banker, 1980; Martinez and Gomes, 2002; Kornyei et al., 2007). In order to investigate if neuronal arborization induced by T3-primed astrocytes was mediated by soluble factors, embryonic cerebral cortex progenitors were cultured in the presence of conditioned medium obtained from control astrocytes (C-CM) or T3-treated astrocytes (T3-CM), for 24 h (Figures 2A,B) and number of neurons quantified after immunostaining for the neuronal marker, βTubulin III. T3-CM had no effect in the number of βTubulin III positive cells compared to C-CM (Figure 2C). On the other hand, T3-CM enhanced neuronal arborization as revealed by a decrease in the number of aneuritic cells (T3-CM: −23%), followed by an increase in those cells with 2 neurites (T3-CM: +58%) (Figure 2D). Likewise, neurite outgrowth was improved by conditioned medium derived from T3-treated astrocytes (Figure 2E). In the presence of T3-CM, most of the neurons presented neurites with average size between 50 and 100 μm, whereas most of those in C-CM exhibited an average size between 10 and 50 μm (Figure 2F). Nevertheless, T3-CM was not able to fully mimic the effects of cocultures on neuronal differentiation and arborization (compare Figures 1, 2), suggesting, a role for astrocyte-neuron contact factor in this process.

### Thyroid hormones-treated astrocytes induce neuronal differentiation and neurite outgrowth through modulation of proteoglycans components

**Figure 3:**
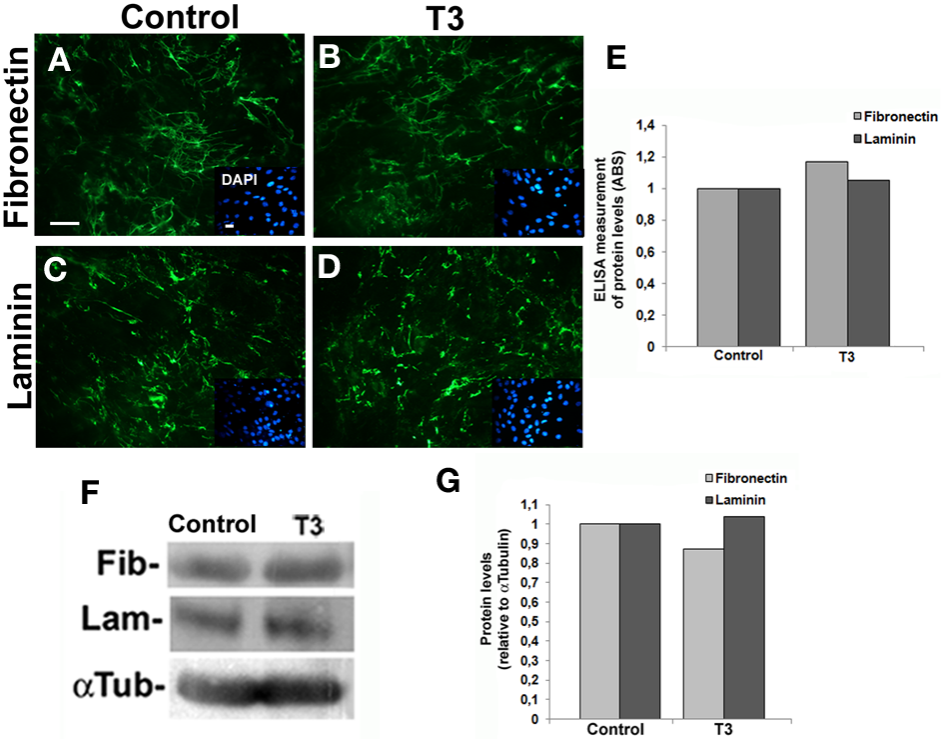
**Thyroid hormones do not affect levels and organization of laminin and fibronectin on cerebral cortex astrocytes.** After reaching confluence, cerebral cortex primary astrocyte cultures were maintained for 3 days in DMEM-F12 medium alone (Control) or supplemented with T3 (T3). Cells were immunostained for Fibronectin **(A,B)** and Laminin **(C,D)**. Thick fibrils characteristics of ECM networks are shown throughout the extracellular membrane surface. DAPI stained nuclei (blue) reveal astrocyte monolayers localized underneath Fibronectin and Laminin matrix (green). No significant differences in the organization and distribution of both ECM proteins were observed **(A–D)**. ELISA assays of total protein extracts from cortical astrocyte cultures, did not reveal any difference in Fibronectin or Laminin levels in control or T3-treated cultures **(E)**. Western blot assays of total protein extracts from cortical astrocyte cultures did not reveal differences in the protein levels after T3 treatment of cortical astrocytes **(F,G)**. Immune reaction for α-Tubulin was used as loading control. Scale bar: 30 μm **(A)**.

ECM proteins are key regulators of neuronal differentiation, migration, axonal projection, neurite outgrowth, synaptogenesis, and regeneration (Faivre-Bauman et al., 1984; Carri et al., 1988; Hammarback et al., 1988; Chamak and Prochiantz, 1989; Martinez and Gomes, 2002). Recently, we demonstrated that astrocytes cultured under hypothyroidism-like conditions, present FN decreased level (Dezonne et al., 2009). Moreover, we also described that thyroid hormone treatment induces FN and LN reorganization in cerebellar astrocytes (Martinez and Gomes, 2002). We then sought to investigate if thyroid hormone treatment affects synthesis and organization of cerebral cortex astrocytic ECM. To accomplish that, the content of LN and FN of cortical astrocytes was evaluated by immunocytochemistry, western blot, and ELISA assays (Figure 3). In all conditions, immunolabeling showed the thick and fibrous networks staining pattern of these two proteins, with no clear difference between control and treated-astrocytes (Figures 3A–D). Equally, neither ELISA nor western blot assays revealed any obvious differences in LN or FN protein levels (Figures 3E–G), suggesting that other components of ECM might be involved in the neurite outgrowth induced by T3-primed astrocytes.

**Figure 4:**
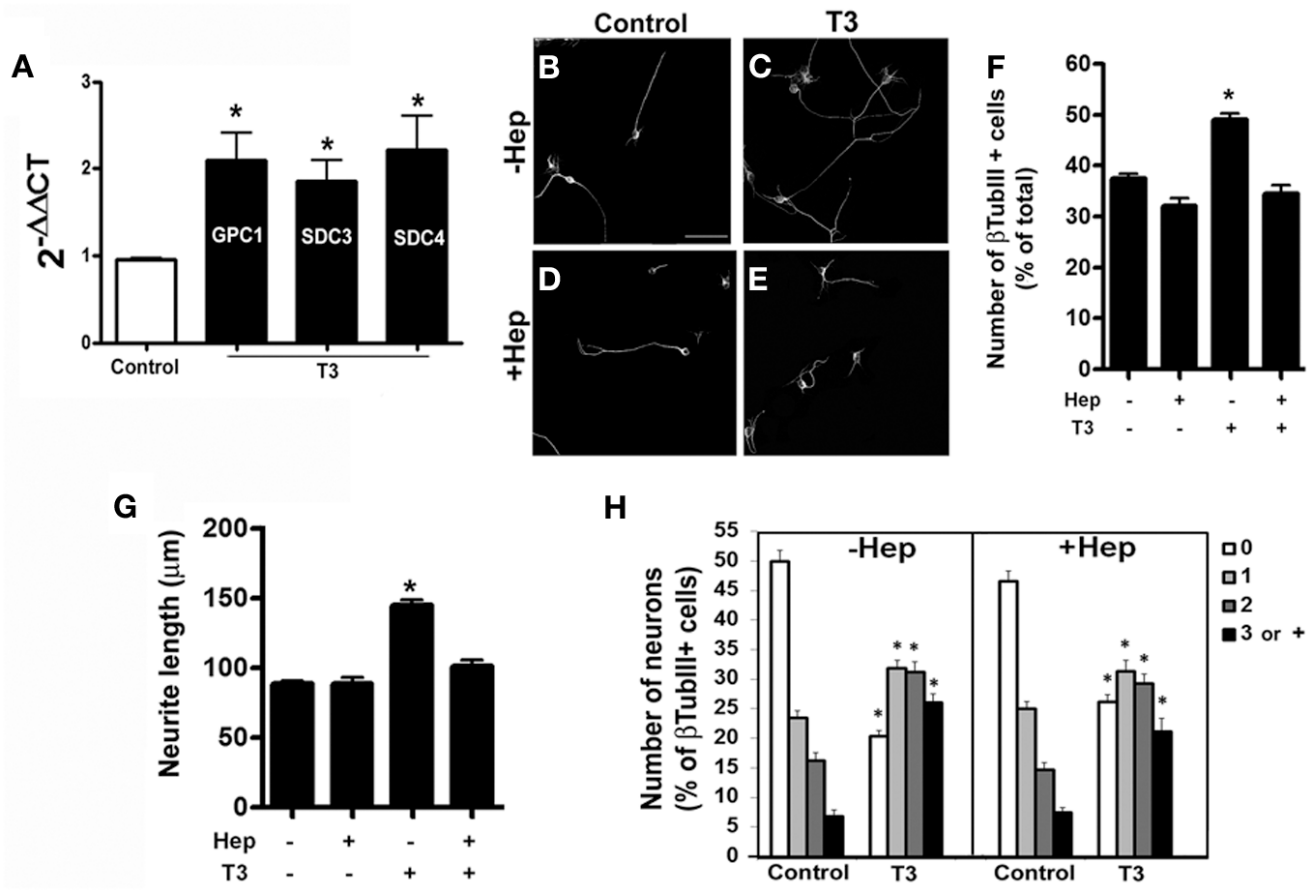
**Thyroid hormone-primed astrocytes induce neuronal differentiation, arborization and neurite outgrowth through heparan-sulfate proteoglycans.** Cortical astrocytes cultures were evaluated for Glypican 1 (GPC1) and Syndecans 3 and 4 (SDC3, SDC4) heparan-sulfate proteoglycans mRNA expression by real time RT-PCR. Representative graph analysis shows that T3 promoted increase in GPC1, SDC3, and SDC4 expression **(A)**. Cortical control astrocytes **(B,D)** or treated with T3 **(C,E)** were enzymatically treated with Heparanase III **(D,E)** previously to addition of embryonic cortical progenitors. After 24 hr of coculture, number of neurons **(F)**, length of neurites **(G)** and number of neurites **(H)** were evaluated. Digestion of heparan chains completely impaired the effects produced by T3 in neuronal number, arborization and neurite outgrowth. Scale bars: 50 μm **(B)**, ^*^P < 0.05.

A large number of proteoglycans have been implicated in regulation of neurite outgrowth. These molecules provide signals that either stimulate or inhibit axonal growth during CNS morphogenesis (Williamson et al., 1996). HSPG are generally correlated with neuronal maturation and neurite outgrowth (Bespalov et al., 2011; Mammadov et al., 2012). In order to investigate if thyroid hormone treatment affects the expression of HSPGs in cerebral cortex astrocytes, we performed a quantitative RT-PCR for the two family of HSPG: syndecan (SDC) and glypican (GPC). T3-primed astrocytes presented an increase of 100%, in the expression of SDC4 and GPC1, and 98% of SDC3 (Figure 4A).

We then sought to analyze the involvement of the HSPG components in cerebral cortex glia-induced neuronal fate commitment and axonal growth. To do that, astrocyte monolayers were treated with heparanase III, which specifically digests HSG chains, after T3 treatment and previously to coculture assays. Enzymatic digestion of HSG chains completely abolished the increase in neuronal population induced by thyroid hormones treatment (Figures 4B–F). It also reversed neurite outgrowth (Figure 4G), but not the increase in neurite number (Figure 4H) induced by T3-treated astrocytes.

**Figure 5:**
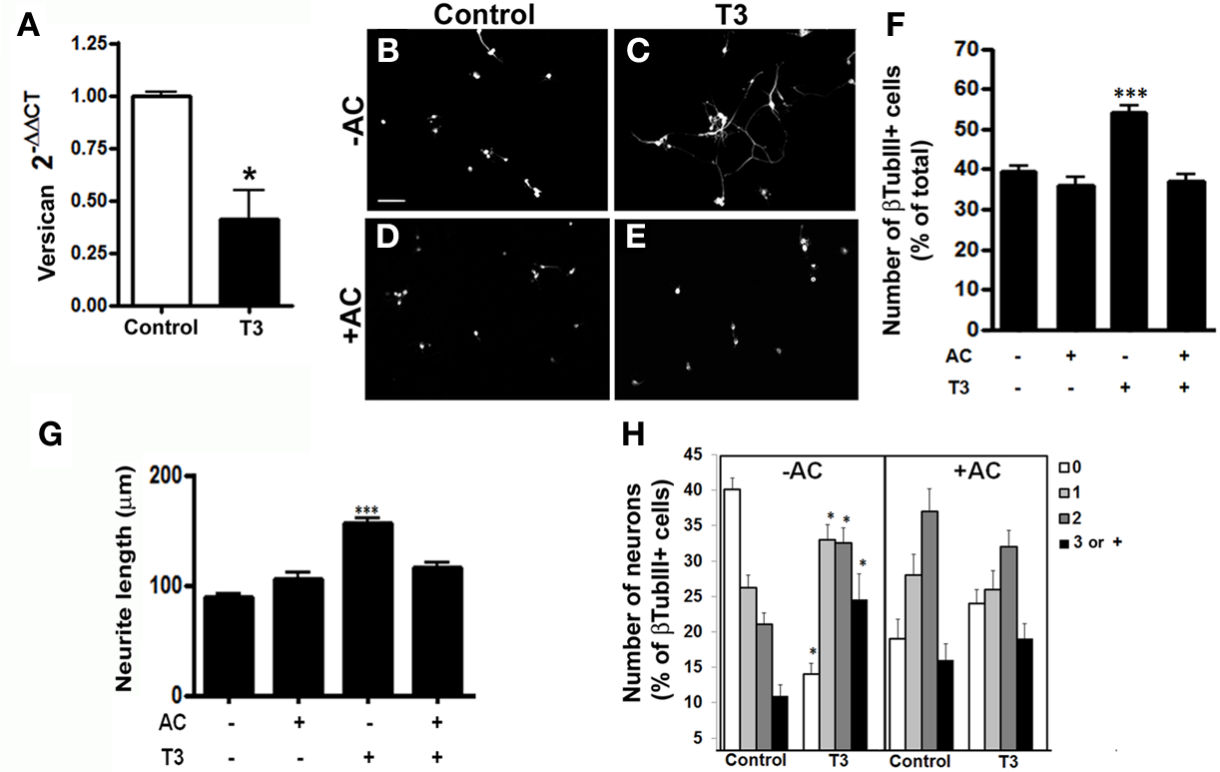
**Thyroid hormone-primed astrocytes induce neuronal differentiation, arborization, and neurite outgrowth through chondroitin-sulfate proteoglycans.** Cortical astrocytes cultures were evaluated for Versican chondroitin-sulfate proteoglycan mRNA expression by real time RT-PCR. Representative graph analysis shows that T3 promoted decrease in the Versican expression **(A)**. Cortical control astrocytes **(B,D)** or treated with T3 **(C,E)** were enzymatically treated with chondroitinase AC **(D,E)** previously to addition of embryonic cortical progenitors. After 24 hr, number of neurons **(B,F)**, length of neurites **(G)** and number of neurites **(H)** were evaluated. Digestion of chondroitin chains completely impaired the effects produced by T3-astrcoytes in neuronal number and neurite outgrowth. Scale bars: 50 μm **(B)**, ^*^P < 0.05; ^***^P < 0.001.

Growing axons navigate complex environments thus, integrating with local structural and chemical cues in order to make net growth decisions. This process is driven by positive and negative clues present in neuronal neighborhood. Among the inhibitoriest molecules to axonal growth are chondroitin sulfate proteoglycans (CSPG) (Nakamae et al., 2009). We thus analyzed the levels of Versican, the major CSPG in the brain and which has been shown to inhibit neurite outgrowth *in vitro* and *in vivo* following injury (Morgenstern et al., 2002). Quantitative RT-PCR assays revealed a 50% decrease in the expression of Versican in T3-treated astrocyte monolayers (Figure 5A).

To evaluate if CSPG might be also responsible for the neuronal maturation induced by T3-treated astrocytes, we enzymatically treated astrocyte monolayers with chondroitinase AC, prior to addition of E14 cerebral cortex progenitors. The digestion of chondroitin sulfate glycosaminoglycan chains completely abolished the increase in neuronal population induced by thyroid hormones treatment (Figures 5B–F). Besides, chondroitinase AC completely inhibited neurite outgrowth induced by T3-treated astrocytes (Figure 5G). Unexpectedly, the absence of inhibitor clues, enhance the extension of neurite by neurons (Figure 5H).

Taken together, these data show that T3 induces differentiation and axonal growth of cerebral cortex neurons by modulation of positive and negative signals: through enhancement of HSPG levels and decreasing versican expression by astrocytes.

## Discussion

Here we report that T3-primed astrocytes induce neuronal maturation and neurite outgrowth of embryonic neural progenitors. These events are mediated by signals derived from HSPG and CSPG, specifically GCP1, SDC3, SDC4, and versican. Our work provides evidence of a novel mechanism, through astrocytes, underlying thyroid hormone effects in cerebral cortex development. The fact that astrocytes highly express several isoforms of TRs makes them strong targets for thyroid hormones action during nervous system development (Carlson et al., 1996; Morte et al., 2004). Our findings support experimental observations that correlate thyroid hormones insufficiencies with clear reductions in axonal and dendritic growth (Eayrs, 1955; Ruiz-Marcos et al., 1979, 1988), and they strengthen the role of astrocytes in these processes, suggesting a thyroid hormone-indirect action on neuronal cells.

Thyroid hormone-conditioned medium also increased astrocyte permissivity to neurite outgrowth, although in less scale than coculture systems, leading to the hypothesis that either astrocyte conditioned medium (ACM) contains a soluble factor with redundant effects of cell contact on these events; or the effects of the ACM are due to soluble proteoglycans. The identity of the putative astrocyte-derived soluble factor should await further investigation.

Congenital hypothyroidism characterized by morphological brain alterations results in disturbed neuronal migration, deficits in axonal projection and synaptogenesis (Eayrs, 1955; Ruiz-Marcos et al., 1979, 1988; Gravel et al., 1990; Berbel et al., 1993; Calikoglu et al., 1996; Cuevas et al., 2005). It is well known that signals derived from ECM are essential to these events, especially those triggered by FN and LN (Carri et al., 1988; Hammarback et al., 1988; Chamak and Prochiantz, 1989; Martinez and Gomes, 2002). We previously demonstrated that thyroid hormones induce neurite outgrowth of cerebellar granular neurons through synthesis and secretion of FN and LN *in vitro* (Martinez and Gomes, 2002). These data contrast with those shown here since we did not observe any alteration in both ECM proteins, either at their levels or organization in the extracellular matrix. These apparent discrepancies might be due to species specificity, since in previous work rats were used and here we used cerebral cortex derived from mice. An alternative possibility is that these differences reflect astrocyte heterogeneity within CNS, since those works analyzed hormone effects in cerebellar development instead of cerebral cortex. It is known that astroglial cells derived from distinct brain regions markedly vary in their responsiveness to thyroid hormone (Lima et al., 1997). It has also been speculated that spatial differences in the expression of T3 receptors account for the variety of T3 response elicited in brain structures (Lima et al., 1997; Gomes et al., 1999).

Another possible explanation is that thyroid hormones exert an indirect action on LN and FN mediated by secondary growth factors secreted in response to hormone treatment. This is supported by the fact that a direct T3-regulation has not been undoubtedly reported for LN and FN, whereas several growth factors secreted by astrocytes in response to thyroid hormone, like EGF and FGF2, have been shown to modulate ECM components (Calloni et al., 2001; Martinez and Gomes, 2002; Mendes-De-Aguiar et al., 2010). Our data agree with those obtained from Farwell and Dubord-Tomasetti who demonstrated that T4, but not T3, increases LN expression (Farwell and Dubord-Tomasetti, 1999a,b).

Enzymatic digestion of glycosaminoglycan chains, with chondroitinases AC and heparanase III, completely abolished the effects of thyroid hormone primed-astrocytes on neuronal phenotype acquisition observed here, although a basal neurite extension was observed in all conditions. Since the optimum concentration of HSPG to elicit neurite outgrowth *in vitro* is unknown, one possibility is that the remaining HSPG, either in control or T3-treated cultures, is sufficient to induce a basal neurite extension. In fact, our data show that both heparan sulfate and chondroitin sulfate glycosaminoglycan chains were important for neurite outgrowth, since we only disrupted glycosaminoglycan chains instead of core proteins. However, it might be related with an alteration in HSPG and CSPG expression by T3-treated astrocytes. In mammalian tissues, most of glycosaminoglycans are covalently linked to proteins, forming the proteoglycans. These macromolecules exert important roles during morphogenesis and homeostasis of the CNS (Carey, 1997; Song and Filmus, 2002; Matsui and Oohira, 2004). Although specific receptors have not been identified for these molecules, they mainly act by interacting with cell adhesion molecules and growth factors (Wang et al., 2008; Mythreye and Blobe, 2009). The CNS presents multiple species of proteoglycans in the ECM and at cell surface, including the two families of HSPG, syndecans and glypicans (Bandtlow and Zimmermann, 2000; Matsui and Oohira, 2004), and many CSPGs, such as neurocan, versican and aggrecan (Carey, 1997; Bandtlow and Zimmermann, 2000; Oohira et al., 2000; Song and Filmus, 2002; Akita et al., 2004; Matsui and Oohira, 2004).

The study of the role of proteoglycans in CNS development and pathology has provided contradictory results regarding their permissive or inhibitory effects on axonal growth. In the adult CNS, CSPGs are upregulated after CNS injury and constitute the main component of the glial scar, which impairs axonal regeneration (Jones et al., 2003; Wang et al., 2008). The 4-sulfated chondroitin chains have been shown to repeal growing axons of several cell types including cerebellar granular cells and dorsal root ganglion neurons (Oohira et al., 2000; Matsui and Oohira, 2004; Wang et al., 2008). However, Wang and co-workers have also shown that in reactive astrocyte monolayers, 6-sulfated chondroitin did not show any inhibitory action on axonal guidance. In addition, ascending sensory axons regenerate into areas where CSPG is expressed after spinal cord injury (Pasterkamp et al., 2001; Inman and Steward, 2003). Versican is an ECM CSPG, and its isoforms are aberrantly expressed in CNS injuries. Diverse works suggest that versican plays key inhibitory role in regulating neurite outgrowth (Zhang et al., 2011). In the present work, we showed that T3-treated astrocytes decreased versican expression, which might contribute to neurite outgrowth.

HSPG are permissive to neurite outgrowth and among them is the transmembrane family of HSPG, SDC. Here, we demonstrated that enhanced expression of SDC 3 and 4 by T3-treated astrocytes is associated with neuronal maturation. These data are in concert with reduced expression of SDC 1, 2, and 4, and increased expression of SDC3 in hypothyroid cerebellum (Mendes-De-Aguiar et al., 2008). Furthermore, a reduced expression of SDC2 and 3 in T3-treated astrocytes has been previously associated with T3 action mediated by FGF2 in cerebellar astrocytes. In this case, proteoglycans were suggested to affect the formation of the trimeric signaling receptor complex composed by SDC/FGF/FGF-receptor, which is essential for FGF receptor dimerization, activation, and cell signaling (Mendes-De-Aguiar et al., 2008). It is known that SDC3 are involved in biological functions such as cell adhesion, cell migration and neurite outgrowth (Akita et al., 2004; Choi et al., 2011). N-syndecan (SDC3) is abundantly expressed in the major axonal pathways and in the migratory routes of the developing brain. They might associate with heparin-binding growth-associated molecule (pleiotrophin), and mediates cortactin-Src kinase-dependent neurite outgrowth (Hienola et al., 2006). In addition, SDC3 might bind to neurocan, a major CSPG expressed by neurons, thus, promoting neurite outgrowth (Akita et al., 2004). Thus, it is not completely unpredicted that CSPG might be permissive to certain neuronal types under peculiar conditions. Besides, SDC family might have a chondroitin sulfate modification, thus, presenting a hybrid class of proteoglycans (Bernfield et al., 1999). Yet, a balance between inhibitor and permissive molecules, rather than individual molecules, might be a determinant factor to neurite outgrowth.

Glypicans are another important family of HSPG. GPCs are linked to extracellular surface of plasmic membrane through a covalent glycosyl-phosphatidylinositol (GPI) anchor. Six GPC were described in mammals so far (GPC1-6). In general, they are expressed predominantly during development when they control tissues morphogenesis (Song and Filmus, 2002). GPC1, the major HSPG of the brain, displays a higher expression level throughout CNS, mainly in the developing neuroepithelium surrounding ventricles (Litwack et al., 1998; Song and Filmus, 2002). Previously works reported that two members of the Slit family, Slit1 and 2, bind to GPC1, and have an overlapping patter of expression in the brain (Ronca et al., 2001). Slits proteins are expressed by neurons and glial cells and play key role in axonal guidance (Brose and Tessier-Lavigne, 2000). In addition, we found an increment of amyloid precursor protein (APP) _m_RNA in T3-treated astrocytes (data not shown). It is well known that SDC3 GPC1 also binds to APP and this interaction promotes neurite outgrowth (Williamson et al., 1996; Clarris et al., 1997; Cheng et al., 2011; Hoe et al., 2012). In this case, neuronal SDC3 and GPC1 might also require astrocyte APP for neurite outgrowth. Here, we showed for the first time that the thyroid hormone, T3, induces neurite outgrowth through upregulating GPC1 expression in astrocyte.

**Figure 6:**
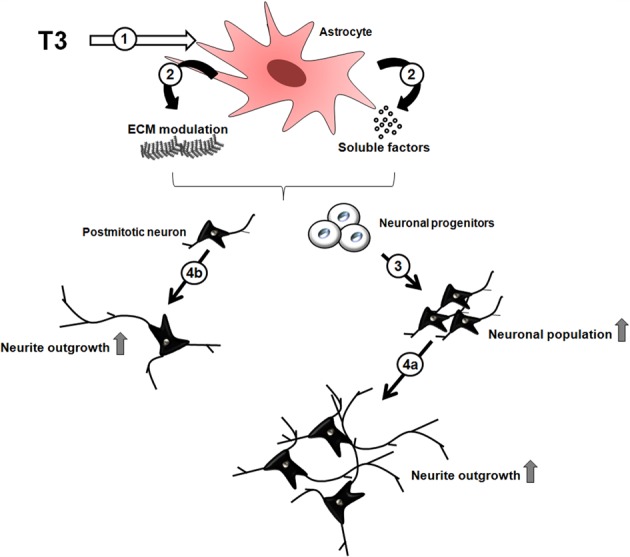
**Indirect effects of thyroid hormone on neuronal differentiation through astrocytes.** The thyroid hormone, **T3**, induces astroglial cells **(1)** to modulate **ECM** molecules expression, such as heparan sulfate and chondroitin sulfate glycosaminoglycans and **soluble factors (2)**, which in turn induce neuronal commitment of progenitor cells followed by their differentiation **(3)**, and maturation with neurite outgrowth **(4a)**. In parallel, postmitotic neurons can be direct targets of astrocyte-derived molecules, thus, promoting maturation and neurite outgrowth **(4b)**.

We demonstrated that thyroid hormone induces neuronal maturation and neurite outgrowth of cerebral cortex neurons *in vitro* and implicated SDC3 and 4, GPC1 and versican produced by astrocytes, in these events. Moreover, we shed light on glial cells as potential targets of thyroid hormone actions during cerebral cortex neuronal development. Upon hormone influence, astrocytes secrete soluble factors, downregulate ECM proteins, specifically, versican CSPG; and upregulate HSPG (SDC3-4, GPC1) thus affecting neuronal pool: either inducing neuronal commitment of progenitor cells and, further, neuronal maturation and neurite outgrowth (Figure 6).

Our work not only increases the knowledge about thyroid hormone role in brain development but helps to identify potential molecules and pathways involved in neurite extension which might eventually contribute to a better understanding of axonal regeneration, synapse formation, and neuronal circuitry recover.

### Conflict of interest statement

The authors declare that the research was conducted in the absence of any commercial or financial relationships that could be construed as a potential conflict of interest.

## Abbreviations

ACM: astrocyte conditioned medium
C-CM: control astrocyte conditioned medium
CM: conditioned medium
CNS: central nervous system
CSPG: chondroitin sulfate-conjugated proteoglycan
D2: type II iodothyronine 5′-deiodinase
DAPI: 4′,6-diamidino-2-phenylindole dihydrochloride
DMEM/F-12: Dulbecco's modified Eagle's medium supplemented with nutrient mixture F-12
E14: embryonic day fourteen
ECM: extracellular matrix
EGF: epidermal growth factor
FBS: fetal bovine serum
FGF: fibroblast growth factor
GPC-1: Glypican 1
HSPG: heparin sulfate conjugated-proteoglycan
PBS: phosphate-buffered saline
SDC-3: Syndecans 3
SDC-4: Syndecan 4
T3: 3, 5, 3′-triiodothyronine
T3-CM: T3- primed astrocyte conditioned medium
T4: Thyroxin
TBS-T: Tris-buffered saline-Tween 20
TRs: thyroid hormones receptors
TRα: isoform α of TR
TRβ: isoform β of TR.
